# Supplementary material for: Chromothripsis during telomere crisis is independent of NHEJ, and consistent with a replicative origin
Source: Genome Res. 2019 May;29(5):737–49. doi: 10.1101/gr.240705.118 (PMC6499312; doi:10.1101/gr.240705.118)
Supplement: Supplemental Material [file supp_gr.240705.118_Supplemental_file_1.zip › contigs/annotated_contigs/DB73/contig.4.DB73_length_723_mean_cov_14.3042876902.docx]

**DB73_length_723_mean_cov_14.3042876902**

```
AATGAAACTGAGTAAGTACAAAACAGCAAAATAGCATTGGCCGCATCCCTCAGGGCCTCACGCACGCACCAGCCACAGCTGGTCTGGCC
 >chr7:75947922-75948149 - E=9e-125 p=1e-02
GCCCTGCCCCATTCCTTGAGCCACAAAATGCTGCAGTGATGCTGGTTTGCAAAGCCCCAGAGCTAGGTCGGGGGATTCAGCATCCCTCC

CGGCCCAGGTCTGGGCCCCCTCCCTACCCCAACCTGGGGGCTCTTGCAG|AGCCTCTCCCCCTCCCTCATCACTTCCTCT|GACGCTTA
                                                                                >chr7:7
GTTGACACTGGTCGTCAGGGAGGGTTTCCTAGAGGAAGTGATGCCTGCGCAGTGTCTTTGGGAAACTTG|AG|CTGGGCATGGTGACGC
5976361-75976440 - E=1e-35                                            >chr7:75968639-75
ACACCTGTAATCCCAGCTACTCGGGAGGCGGAGACAGGAGAACTGCTTGAACCCGGGAGG|CA|ATCAATTCTAGTATTTTTCCAGTTT
968719 + E=2e-36                                             >chr7:75969539-75969850 +
ATTCTCTACAAATATAACCTATGGTTTCCAAATATCACCTGTCCTTTCCCATAGTTACAATTTCCTATACTTGAATCATATACTACCTG
E=3e-175
GACAAGGCCAATGCCAATAATGCTTATGAGCATTTTTTTGTTTTGTTTTGAATTCAATGGAACTATTTCCAGTGGTTTCATTCTTAAAT

GTCAGCTCTTGGTTTAATTGTATTTTTCTCTATCATGTTAAGAAAGTATCCATTTAGTCCATATTTTTCTAATAAACTTTTGTGATCAG

AAATGGATTTTTCAACA
```
